# GOARN training: supporting field epidemiology trainees to upskill for public health emergency response

**DOI:** 10.5365/wpsar.2024.15.5.1102

**Published:** 2024-04-22

**Authors:** Yasmin Lisson, Keeley Allen, Tony Stewart, Amy Elizabeth Parry

**Affiliations:** a Australian Government Department of Health and Aged Care, Woden, Australian Capital Territory, Australia.; b Department of Applied Epidemiology, National Centre for Epidemiology and Population Health, The Australian National University, Acton, Australian Capital Territory, Australia.; c Health Protection Services, ACT Health, Holder, Australian Capital Territory, Australia.; * These authors are equal contributors to this work and are designated as co-first authors for this article.

Global health security is underpinned by a skilled public health workforce. Epidemiologists play a crucial role not only in protecting individuals and communities from infectious diseases and public health events, but also in establishing relationships among countries and areas in the Western Pacific Region. (*1*) Field epidemiology training programmes (FETPs) build and strengthen a nation’s capacity to respond to health threats both locally and regionally by providing graduates with technical expertise in public health surveillance, outbreak investigation, risk assessment and data analysis. To be impactful as epidemiologists in public health emergency responses, training such as the multitiered Global Outbreak Alert and Response Network (GOARN) training course, specifically the introduction to emergency response tier of the programme (Tier 1.5), is essential to the curriculum. GOARN Tier 1.5, which incorporates the online modules of Tier 1 learning, is an important supplement to the FETP curriculum, providing simulation exercises for public health emergencies that fellows may not experience in their field placements.

The Australian advanced FETP is located within the National Centre for Epidemiology and Population Health (NCEPH) at the Australian National University (ANU). (*2*) NCEPH has been a GOARN partner since the network’s inception in 2000 and has maintained a close relationship with the GOARN team. An opt-in GOARN Tier 1.5 training has been conducted with Australian FETP trainees on an ad hoc basis on five occasions over several years to increase the pool of field epidemiologists ready to deploy for international public health emergency response. However, due to the broad-reaching impacts of the COVID-19 pandemic, for the first time the Australian FETP took a systematic approach and extended the Tier 1.5 training to all trainees of the 2021 and 2022 cohorts, which provided them with an introduction to international outbreak response. Australian FETP trainees could then apply individually for further tiers, if interested. This article outlines our perspective on the importance of upskilling FETP trainees for public health response.

In September 2022, over 30 Australian FETP trainees participated in a GOARN Tier 1.5 training tailored for field epidemiologists. The training consisted of two components: a series of self-directed online modules (Tier 1) and a face-to-face workshop (Tier 1.5) (**Table 1**). The online modules covered GOARN’s core objectives, the structures of an international response with GOARN and the World Health Organization, information regarding pre-deployment processes, safety and cultural awareness in the field, and some of the challenges involved in fieldwork in low-resource and remote settings. The face-to-face workshop encouraged further reflection through in-depth group discussions and interaction through real-world scenario-based situations. Australian FETP trainees finished the training with a deeper understanding of the realities of deployment, multidisciplinary teamwork, leadership, capacity-building and best practices for working with local communities, governments and partners during a domestic or international public health emergency response.

Peer-to-peer learning is a powerful tool used during FETP training and is also a critical element of the Tier 1.5 training. Real-world scenario discussions used in the training showcased the complexities of working in high-pressure environments as well as rapidly changing and time-critical situations. The personal stories shared by former deployers were thought-provoking and included wide-ranging examples, from expanding clinical capacity and infection control standards in low-resource settings to problem-solving for pregnant women with Ebola virus disease. At the heart of these responses were well equipped and prepared responders, applying agile thinking and intercultural fluency when working with communities and with their emergency response team members. Participation in the training enabled FETP trainees to learn directly from practitioners who had deployed to a range of different international emergency response settings and provided an opportunity for them to ask questions and seek clarifications.

Critically, the training imparted the importance of interpersonal skills such as communication and collaboration, sometimes known as “field skills.” Field skills are essential skills for all responders, whether deployed domestically or internationally; however, research has found that applied epidemiologists deployed to public health emergency responses have reported challenges with interpersonal communication, working within multicultural, multidisciplinary teams, and clarifying the role of an epidemiologist. (*3*, *4*) Understanding these nuances and developing these capabilities are necessary for a successful public health emergency response and may not be addressed in the current FETP curriculum. (*5*, *6*)

FETPs focus on developing technical skills and applied epidemiological methods skills among its trainees. Despite the training being mostly based in the “field” (workplace), a trainee is likely to progress through their FETP without exposure to an international public health emergency response. (*6*) Depending on their workplace and the projects they undertake as part of the programme, field skills such as communication and leadership may or may not be developed. (*3*, *6*) FETP trainees are therefore unlikely to test their technical and field skills in a real-time emergency, within a supportive learning environment.

Indeed, despite the breadth of knowledge and expertise among Australian FETP trainees at the time of the GOARN Tier 1.5 training, few had experience in international public health emergency response. The training provided a safe place for trainees to explore and self-reflect on their skills, personality and individual career objectives, and the skills they needed to hone before international deployment. Further, the training highlighted important considerations that are transferrable to domestic public health emergency response, including communication strategies across multidisciplinary teams and the need to clarify roles and responsibilities during the response.

During public health emergency response, field epidemiologists play a pivotal role in mitigating adverse impacts on communities. To enhance the pool of well prepared deployers, collaboration between FETPs and GOARN is essential. This collaboration ensures that FETP trainees possess the necessary skills for effective domestic and international responses. (*6*, *7*) The COVID-19 pandemic exposed the fragility of global health systems and underscored the need to strengthen national workforces and broaden the regional network of responders. Now more than ever, the public health workforce needs FETP trainees and alumni to be better able to apply both their technical and field skills across various contexts to drive positive health outcomes. The Australian FETP aims to continue its partnership with GOARN through routine training, mentored field deployments, and upskilling FETP faculty to complete GOARN training of trainers. The GOARN Tier 1.5 training provided a valuable learning experience for the Australian FETP trainees and should be considered as an ongoing component of the curriculum of advanced FETPs internationally.
